# A Fuzzy Inference System for the Assessment of Indoor Air Quality in an Operating Room to Prevent Surgical Site Infection

**DOI:** 10.3390/ijerph19063533

**Published:** 2022-03-16

**Authors:** Ylenia Colella, Antonio Saverio Valente, Lucia Rossano, Teresa Angela Trunfio, Antonella Fiorillo, Giovanni Improta

**Affiliations:** 1 Department of Electrical Engineering and Information Technologies, University of Naples “Federico II”, 80125 Naples, Italy; ylenia.colella1@gmail.com (Y.C.); antoniosaverio.valente@unina.it (A.S.V.); lucia.rossano2020@gmail.com (L.R.); 2 Department of Advanced Biomedical Sciences, University Hospital of Naples “Federico II”, 80131 Naples, Italy; antonellafiorillo@virgilio.it; 3 Department of Public Health, University of Naples “Federico II”, 80131 Naples, Italy; ing.improta@gmail.com; 4 Interdepartmental Center for Research in Healthcare Management and Innovation in Healthcare (CIRMIS), University of Naples “Federico II”, 80131 Naples, Italy

**Keywords:** fuzzy logic, indoor air quality, operating room, surgical site infection

## Abstract

Indoor air quality in hospital operating rooms is of great concern for the prevention of surgical site infections (SSI). A wide range of relevant medical and engineering literature has shown that the reduction in air contamination can be achieved by introducing a more efficient set of controls of HVAC systems and exploiting alarms and monitoring systems that allow having a clear report of the internal air status level. In this paper, an operating room air quality monitoring system based on a fuzzy decision support system has been proposed in order to help hospital staff responsible to guarantee a safe environment. The goal of the work is to reduce the airborne contamination in order to optimize the surgical environment, thus preventing the occurrence of SSI and reducing the related mortality rate. The advantage of FIS is that the evaluation of the air quality is based on easy-to-find input data established on the best combination of parameters and level of alert. Compared to other literature works, the proposed approach based on the FIS has been designed to take into account also the movement of clinicians in the operating room in order to monitor unauthorized paths. The test of the proposed strategy has been executed by exploiting data collected by ad-hoc sensors placed inside a real operating block during the experimental activities of the “Bacterial Infections Post Surgery” Project (BIPS). Results show that the system is capable to return risk values with extreme precision.

## 1. Introduction

In hospitals, airborne particles can be a serious threat to patients, as, it is known that most opportunistic pathogens that cause hospital-acquired infections (HAIs) are at least partly dispersed in the air [1], especially in the operating department (OD) [2]. Their presence, in fact, could be a source of infections such as those of the surgical site. To limit the onset of contamination, an OD is typically divided into progressively less contaminated areas, from the entrance to the operating theatres. The operating room (OR) is a special unit requiring a clean environment, with the fewest number of particles in the air. For this reason, OR contemplate internal paths, differentiated into “dirty” and “clean”, for the safe collection and transport of materials and the correct personnel’s dressing. In addition, during surgical procedures, the team and the surrounding environment release dust particles, textile fibers, and respiratory aerosols loaded with vital microorganisms [3,4,5], that can settle on surgical instruments or enter directly into the surgical site causing surgical site infections (SSI) [6]. Indeed, different methodologies have been used to define, measure, analyze, improve, and control the onset of healthcare-associated infections that compromise patient safety [7]. Nowadays, the monitoring and prevention of healthcare-associated infections (HAIs) are a priority for the healthcare sector [8,9]. The SSI are the most common type of HAI, contributing to significant morbidity, costs, and deaths [10]. Badia et al. confirmed that patients with SSI required prolonged hospitalization, reoperation, readmission, and increased mortality rates, compared to uninfected patients. Therefore, SSI were consistently associated with high costs [11].

Therefore, to reduce the SSI-related mortality rate, airborne particles need to be monitored. A fundamental role is played by the heating, ventilation, air conditioning (HVAC) system, those systems provide ventilation capable of maintaining temperature and humidity at optimal levels, but also of keeping the concentrations of airborne gaseous pollutants, particles, and microbes below predetermined levels [12].

In this paper, we propose an operating room air quality monitoring system based on fuzzy logic (FL). Due to the unclear (fuzzy) nature of medical data and models, as well as the relationships that exist in the models, the FL technique is particularly used in the healthcare context. Indeed, Owoseni et al. developed a fuzzy system that diagnoses malaria uncertainties as expected from a human expert in the field of medicine [13]. Furthermore, Leite et al. presented a fuzzy model capable of monitoring and classifying the vital sign’s condition of hospitalized patients, sending alerts based on the pre-diagnosis made helping the medical diagnosis [14]. Fuzzy expert systems (FES) can help support doctors’ decision making, based on complex and uncertain parameters that play a crucial role and require scrupulous evaluations [15,16,17]. For example, these systems could help doctors prevent, diagnose, and treat disease [18,19]. Air quality control was also included in the scope of FL, in the interior of buildings and hospitals, as shown in the literature [20]. Kumar et al. [21] designed a fuzzy logic controller (FLC) to ensure optimal conditions for the OR. In this work, we propose to exploit temperature, humidity, particles, and oxygen as input parameters of an FLC to regulate the level of fresh air and fan circulation as output parameters in order to restore air quality to optimal levels. Instead, Aggarwal et al. [22] proposed a fuzzy interface system to calculate the air quality index using two pollutants (PM2.5 and PM10) considering six linguistic variables for each in order to monitor pollution in real time, obtaining satisfactory results. Dionova et al. [23] proposed an air quality index (EIAQI) based on the environmental indoor air pollutants (IAP) and on four thermal comfort pollutants (TCP). The proposed fuzzy inference system identifies, classify, and evaluates the index based on the value assumed by IAP and TCP. The calculated value of EIAQUI was then exploited to control directly the inlet and outlet exhaust air fan, buzzer, and LED. To solve the problem of indoor air quality in rooms with natural ventilation, Kulis and Müller [24] presented an electronic device equipped with an FLC, based on the air parameters measured with sensors, that recommended opening the windows showing it on the display. This system ensured adequate indoor air quality. Definitely, FL is a methodology that is exploited to monitor the air quality inside buildings in a wide range of the literature. Grychowski group designed a system to monitor indoor environmental conditions, with a special consideration regarding the comfort of occupants, while Talaz group evaluated the indoor air quality in classrooms and offices, using a fuzzy system [25,26].

In this work, we propose a fuzzy inference system (FIS) allowing evaluation of the air quality in the operating room that takes into account several parameters. Compared to other literature works, the proposed approach was designed to take into account also the movement of personnel, the particle count in the operating room (ISO 5, ISO 7), differential pressure, air changes per hour, temperature, and relative humidity. All the presented features have been collected in real time through hi-tech sampling devices and developed directly on the field, through different phases (data collection and adjustment, development of the FIS and its validation on real scenario) by an interdisciplinary research team comprising doctors, clinicians, IT engineers, and an economist. The FIS, obtained by combining the potential of FL and Expert Systems, gives the possibility to monitor OR conditions by appropriately modeling input data and by reproducing the cognitive process of experts through inferential techniques [27].

The goal of the proposed monitoring strategy is to better manage contamination in a surgical environment to prevent SSI—thereby reducing the related mortality rate—and help hospital staff responsible to restore HVAC efficiency. This analysis finds important application results if it considers the impact of the COVID-19 pandemic on healthcare structures. In order to better manage SARS-CoV2 and protect both healthcare personnel and public health, the analysis of contamination risk in operating rooms can have a very concrete aspect in daily hospital practice [28].

Despite the availability of a number of studies examining the issue of aerobiology, there are no studies in the literature applying an FIS to prevent SSI; for this reason, our study presents an innovative key to understanding risk management in hospitals through the support of expert systems.

## 2. Materials and Methods

### 2.1. Context

In order to design and test the proposed FIS, the “Umberto I” Hospital situated in Nocera Inferiore, Salerno (Italy) was chosen. The multi-specialized Operating Department (OD) of the “Umberto I” Hospital is structurally equipped with apposite dirty—clean paths for healthcare personnel, patients, surgical instruments, and waste. The block has a filter zone for patients and healthcare professionals and it is provided with an internal sterilization local.

### 2.2. Experimental Section

**Figure 1:**
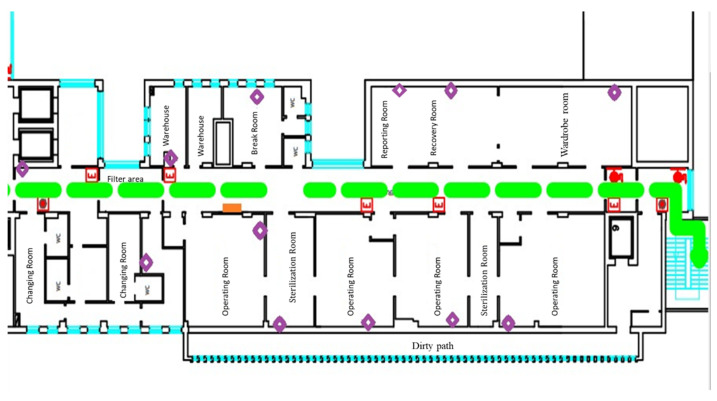
Location of sensors in the OD (Rhombus indicate the EchoBeacon position).

Different sensors were placed inside the operating block during the experimental activities of the BIPS Project in order to collect clinical data (Figure 1).

BLE devices have been installed for monitoring the paths taken by patients and health professionals during ordinary work. In addition, to assess the number of particle charge during the day, a particle counter and a multi-parameter unit allowed monitoring of the microclimatic parameters that have been positioned in OR.

The family of BLE devices that has been chosen for the experimental phase is BluEpyc, consisting of three components:

- Gateway: it is a small device, with a web server, CPU, and I/O card; with a reading range of up to 100 m, able to receive and manage data from the EchoBeacons via Ethernet, Wi-Fi, or GPRS.
- EchoBeacon: also called Reader, it is a signal repeater that allows building BLE configurations in rather large internal areas, within which detailed data acquisition and easy installation are required. The goal of EchoBeacon is to receive data from the Beacons and forward them to the Gateway, through wireless communication. The coverage range for sending and receiving signals is about 100 m.
- Beacon: they are low-cost connectionless devices to fix on objects or wear, used to implement the micro localization and to reveal a change of position of people and objects.

The gateway is the only BLE device connected to the LAN network of the hospital that is able to communicate and exchange information with the main computer in the technical office, on which the BluEpyc software is installed. 

The purpose of BLE devices using is to verify the behavior of staff during surgical interventions. The main non-conformity that has been found during normal activity has been the excessive movement in/out of clinical personnel from the operating room; consequent opening of the doors which produces an increased risk of particle contamination of the air inside the room. It is known that a person sitting on a chair without moving their limbs generates particulate matter around them. Therefore, any movement generates a greater quantity of dust than in a closed environment such as that of an operating room it spreads rapidly throughout its volume. Therefore, the movement of clinical personnel inside the rooms has to be taken into consideration in order to reduce any movements that will involve excessive increases in airborne particulate. The main means of transport for microorganisms contained in the air is precisely the same particulate matter present in the environment. The greater the particulate matter, the greater the chances of having a high bacterial load in the air. The UNI EN ISO 14644-1 standard provides a classification of the operating rooms and the relative required levels of sterility according to the type of interventions performed. The rooms are therefore classified into different categories: ISO 5 for very complex and high-risk specialist interventions, ISO 7 for interventions without material implantation but requiring high protection (Anon., 2016). The operating block of the “Umberto I” Hospital is equipped with four operating rooms classified as ISO 5. Among the equipment installed in the operating block, to better understand the particle concentration in the rooms, a particle counter has also been installed. It was the SOLAIR 3100 that can store up to 3000 records of particle count data from up to 8 particle channels and the configurable recipe database can store up to 50 recipes for sampling and reports. The particle counters have been positioned in all operating rooms in order to collect data in different environmental conditions and to better understand all events that could alter the particles present in the various operating rooms. 

Finally, the last installed measurement device consists of a multi-parameter tool whose purpose is to monitor the climatic conditions of the operating room by measuring the relative humidity, differential pressure, and speed of the air flow in correspondence with the ventilation outlet.

#### Data Input

In the following tables (Table 1, Table 2 and Table 3), the data model collected by each device is reported.

### 2.3. Fuzzy Inference System

A fuzzy inference system (FIS) is an intelligent system that allows reproducing the ability of the human mind to approximate vague data, extracting from them useful information, and producing crisp output [29]. Its potential can be applied to numerous domains and particularly to the medical field to model the high complexity and uncertainty that characterizes medical processes [30,31]. Starting from the definition of the system knowledge base, built through the help of doctors and clinical experts, an FIS gives the possibility to transfer human and expert knowledge into intelligent and automatic models using linguistic terms [32]. Fuzzy sets are used to treat uncertainty and to represent knowledge through rules. The fuzzy logic allows the interpretation of data with predefined linguistic variables according to appropriate IF-THEN rules written as:

IF situation THEN conclusion

where the situation represents the antecedent or the premise consisting of fuzzy terms connected by fuzzy operators, while the output is called consequent or conclusion [33]. Fuzzy logic defines the inferential mechanisms needed for reaching the output value related to the OR’s air quality status starting from its main parameters and constitutes the inferential engine of the FIS.

#### 2.3.1. Knowledge Representation

A linguistic variable is a variable whose values are words or sentences of a language, natural or artificial, exploited to ease a gradual transition between the two states of binary logic and to express in the most natural way the measurements’ vagueness, which is not possible by using crisp variables [29]. Hence, it holds:

**Definition 1.**  *Linguistic variable [33]. A linguistic variable can be characterized by a quintuple (L, F(L), U, R, M) in which L is the name of the variable; F(L) is the term-set of L, that is the collection of its linguistic values; U is a universe of discourse; R is a syntactic rule that generates the terms in F(L); M is a semantic rule which associates to each linguistic value X its meaning, M(X), where M(X) denotes a fuzzy subset of U.*

**Definition 2.**  *Fuzzy variable [33]. A fuzzy variable is characterized by a triple (L, U, F(L; u)), in which L is the name of the variable; U is a universe of discourse (finite or infinite set); u is a generic name for the elements of U; and F(L; u) is a fuzzy subset of U which represents a fuzzy restriction on the values of u imposed by L. F(L; u) will be referred to as the restriction on u or the restriction imposed by L. The assignment equation for L has the form:*

*x* = u∶F(L) (1)

*and represents an assignment of a value u to x subject to the restriction F(L).*

In the universe of discourse U, a fuzzy set F (L; u) is characterized by a membership function (MF) µ(F) that assigns a membership value to elements u, within a predefined range of U, as follows: F = {(u, µF)|u ∈ U and µF:U → [0,1]}. Therefore, a membership function is a distribution that maps every single point of the input space (i.e., the universe of speech, which represents the set of linguistic variables) in a membership value between 0 and 1. For modeling the linguistic variables and the associated membership functions, here we follow the approach proposed by Garibaldi et al. [34]. Accordingly, the membership functions related to the various linguistic variables are composed in order to constitute an appropriate IF-THEN rule.

#### 2.3.2. Fuzzy Inference Engine

All the designed clinical linguistic variables, membership functions, and rules have been included in a Mamdani FIS [35,36]. The Mamdani approach has been widely and successfully applied to different areas, such as data classifications, decision analysis, expert systems, and it is often exploited for designing decision support systems thanks to its ability to imitate human thought processes in complex circumstances and to accurately perform repetitive tasks [37,38]. In the following, the Mamdani framework and the basic knowledge implemented into the system are described with reference to multi-inputs and single-output decision model, and the following definition holds.

**Definition 3.**  *Given m “if antecedent then consequent” fuzzy rules R = {R1; …; Rm}, with n continuous input variables u_i_, i = 1, …n, and the output variable y, the formulation of the fuzzy rules is defined as follows:*

*if*(*u*_1_,*A*_1,1_)*AND*(*u*_2_,*A*_1,2_)*AND*…*AND*(*u_n_*,*A*_1,*n*_)*THEN*(*y*,*B*_1_)
*…* *if*(*u*_1_,*A*_*m*,1_)*AND*(*u*_2_,*A*_*m*,2_)*AND*…*AND*(*u_n_*,*A_m,n_*)*THEN*(*y*,*B_m_*) (2)

*where u^i^ are the input variables, y is the output variable, and Aij and Bi are fuzzy sets of the associated universes of discourse.*

To perform inference, the first step is to “evaluate the antecedent”, which involves fuzzifying the inputs and applying any necessary fuzzy operators to each rule in R as follows.

**Definition 4.**  *Given the information input u = {u1, …,un}, the strength level or firing level αi of the rule Ri is calculated in terms of the degrees of membership µAij. If the antecedent clauses (the if part) are connected with AND then:*

*α_i_* = *min*(*µ*_*Ai*,1_ (*u*_1_), …, *µ_Ai,n_* (*u_n_*)) (3)

*Else if the antecedent clauses are connected with OR then:*

*α*_i_ = *max*(*µ_Ai,_*_1_(*u*_1_), …, *µ_Ai,n_*(*u_n_*)) (4)

*Note that each fuzzy rule yields a single number that represents the firing strength of that rule [39].*

The second step is the so-called “implication,” or, in other words, applying the result of the antecedent to the consequent. Indeed, the strength level is then used to shape the output fuzzy set that represents the consequent part of the rule. In so doing, we have:

**Definition 5.**  *The operator of implication for the rule Ri is defined as the shaping of the “consequent” (the output fuzzy set), based on the “antecedent”. The input of the implication process is a single number given by the “antecedent”, and the output is a fuzzy set:*

*µ_Bi_(y) = min(α_i_(u), µ_Bi_(y))* (5)

*where y is the variable that represents the support value of output the membership function µBi(·).*

Now, in order to unify the outputs of all the rules, we need to aggregate the corresponding output fuzzy set into one single composite set. The inputs of the aggregation process are represented by the clipped fuzzy sets obtained by the implication process. The aggregation method exploited in our application is the max(·) one. 

Finally, the defuzzification process has been performed starting from the output fuzzy set resulting from the aggregation process according to the following definition.

**Definition 6.**  *The operations of defuzzification are computed as the centre of gravity (COG) of the strength levels:*

(6) $$COG\left(y\right)=\frac{\sum_{i=1}^{m}y{µ}_{B_{i}}\left(y\right)}{\sum_{i=1}^{m}{µ}_{B_{i}}\left(y\right)}$$

#### 2.3.3. FIS Implementation

**Figure 2:**
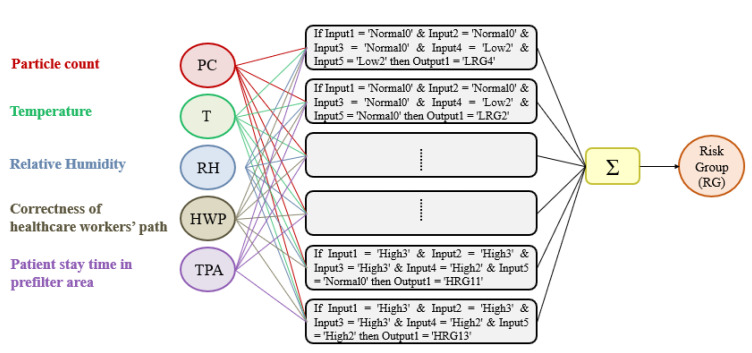
Schema of the fuzzy inference system designed.

The goal of the FIS is to predict the different grades of severity related to OR’s air quality and, hence, synthesize them on a colored graph for ease of representation. The design of the FIS can be summarized into 3 main stages: fuzzification, inference, and defuzzification. All the designed clinical linguistic variables, membership functions, and rules have been included in a Mamdani FIS [40,41]. The Mamdani linguistic model is built on fuzzy IF-THEN rules where both the preceding and consequent sentences contain linguistic variables (Figure 2). Therefore, it is an intuitive model thanks to its ability to implement human knowledge and human experience into the system, as carried out in this work where the knowledge base is determined from the application of “Guidelines on occupational safety and hygiene standards in the operating department” [42,43].

#### 2.3.4. Identification of the Fuzzy Sets

Before their design, there is the need for a preliminary phase for the correct definition of the ranges into which the input variables values must be divided and the choice of the fuzzy sets to be used. The preliminary design phase aims to characterize the inputs and determine the degree to which each of them belongs to a particular fuzzy set through the membership function’s definition. The parameters related to the OR hat will be taken into consideration will be: particle count (PC), temperature (T), and relative humidity (RH). The other two parameters considered are: correctness of healthcare workers’ path (HWP) and time spent by the patient in the prefilter area (TPA). The fuzzification process for each of the listed inputs will be presented below. Finally, the Google Colaboratory platform has been used to perform the fuzzy inference process. Colab uses Python as a programming language and includes most of the standard libraries usually used, including the one dedicated to fuzzy logic.

- Particle count

**Figure 3:**
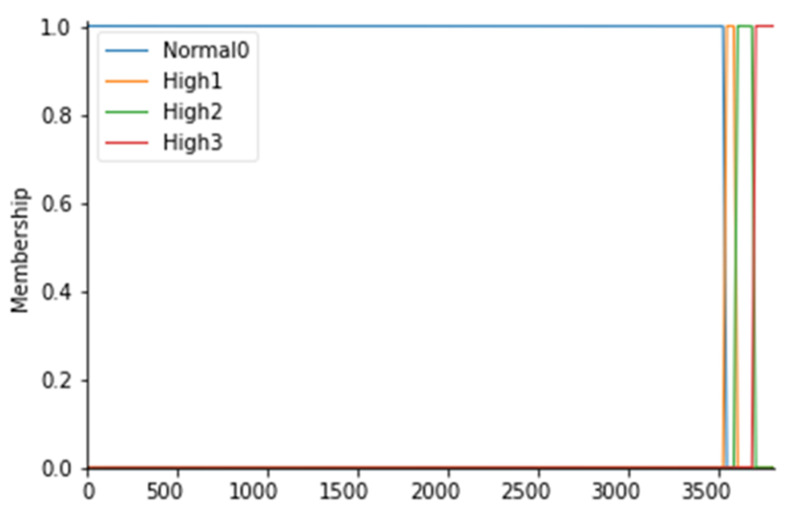
PC membership functions.

For the particle count values, 4 fuzzy sets have been considered, as follows: Normal0, High1, High2, and High3; the membership functions of the 4 fuzzy sets were considered to have a trapezoidal shape. To fuzz the particle count (PC), it is first necessary to identify which values are commonly and unquestionably considered normal for ISO 7 5 operating rooms: this range coincides with values below 3.52000 particles/m^3^ for particles with a diameter equal to or greater than 0.5 µm (Anon., 2016). Consequently, a fuzzy set called Normal0 was created with CP values lower than 3.540 particles/m^3^ with a membership function set to 1 in correspondence with values lower than 3.520 particles/m^3^. In fact, to say that the MF is equal to 1 means to affirm that there is a certain belonging of those values to the fuzzy set Normal0. To create a trapezoidal membership function, however, it is necessary to “blur” the set considered by identifying those values outside the range that can be considered normal to a certain degree, as they could also be an alarm indication. For this reason, a decreasing ramp was considered between the values of 3.520 and 3.540 whose purpose is to gradually smooth the degree of belonging to these values to the Normal0 set with degrees of belonging ranging from 0 to 1. Proceeding in the same way, values above 3.520 particles/m^3^ were identified as high and they were considered as belonging to the fuzzy set called High1 with MF set to 1, identifying the range between 3.520 and 3.600 particles/m^3^ as a transition. Similarly, we proceeded to fuzzify the CP considered even higher up to the fuzzy set High3 for values higher than 3.680 particles/m^3^. Table 4 shows the CP ranges considered with the respective fuzzy sets. The membership functions are instead shown in Figure 3.

- Operating room temperature

For this input parameter, 3 fuzzy sets have been considered: Low2, Normal0 and High2. The membership functions of the 3 fuzzy sets will be trapezoidal.

**Figure 4:**
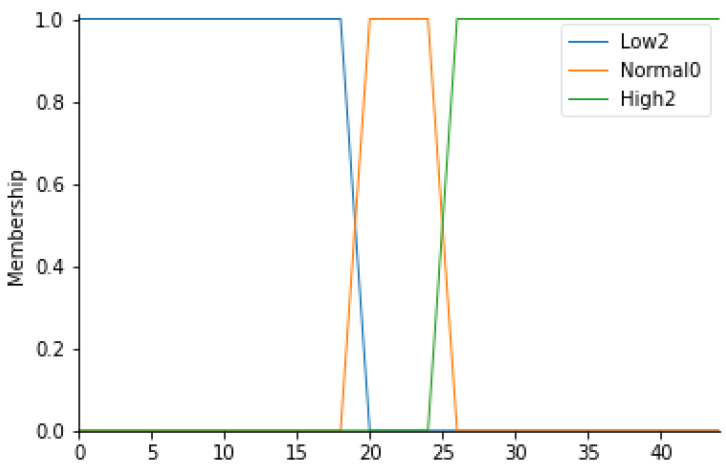
T membership functions.

Following the same procedure for the definition of the ranges for the particle count, it is possible to create a similar table (Table 5) in which the temperature values (T) for each fuzzy set can be displayed. Figure 4 represents the overall membership function.

- Relative humidity

**Figure 5:**
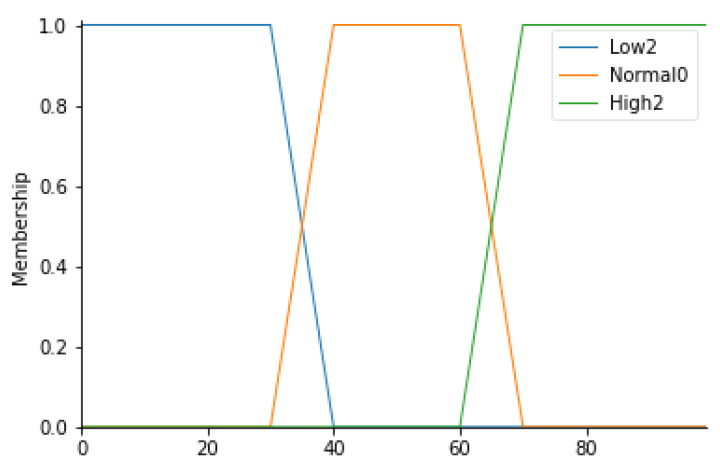
RH Membership functions.

This parameter, expressed in percentage terms, indicates the relative humidity value of the air. A measurement of this parameter in the standard usually ranges between 40% and 60%. In light of this, 3 linguistic variables have been identified: Low2, Normal0, and High2. All values between 30% and 70% are given the Normal0 label with a trapezoidal membership function. Table 6 defines the ranges corresponding to each fuzzy set; Figure 5 shows the corresponding trapezoidal membership functions.

- Correctness of healthcare workers’ path

**Figure 6:**
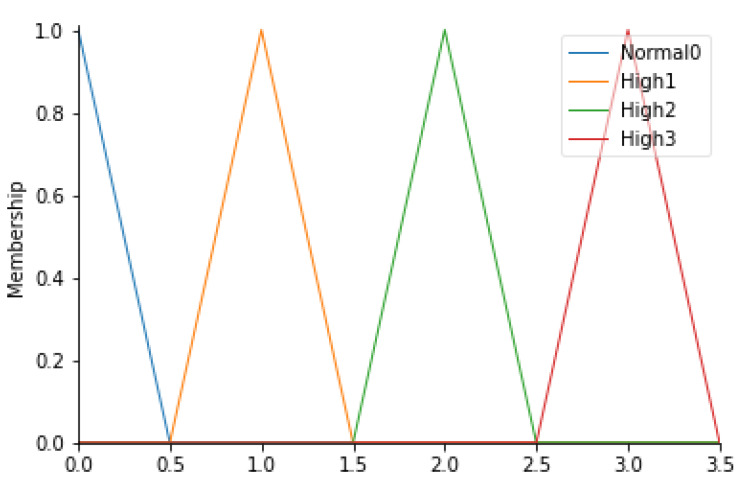
HWP membership functions.

For this parameter, 4 fuzzy sets (Normal0, High1, High2, and High3) have been identified, defined in Table 7. In detail, since these values are an output of an algorithm capable of evaluating the correctness of the path taken by healthcare professionals (HWP), triangular membership functions were considered. Figure 6 shows the corresponding membership functions.

Specifically, a special algorithm was implemented to study the data derived from the detection of the HW equipped with a beacon when they enter into a specific area of the operating block. The dataset is therefore composed of the date and time of detection, user id, area in which the EchoBeacon—that detected the signal—is located, and the RSSI. The algorithm read all the information and was saved on an Excel sheet. Before the realization, starting from the planimetry of the operating block, the possible paths, characterized by minimal movements from one area to another (starting point—final point), were studied. For each path, it is possible to define a level of error. Table 8 shows the paths analyzed with the evaluation expressed according to the fuzzy set.

All paths not shown in Table 8 are considered adequate ‘Normal0’. The analysis was conducted using Python, a high-level interpreted programming language. The openpyxl is a Python library that reads Excel sheets. In order to evaluate the correctness of the path, since the areas in which a specific HW is located, a matrix has been built that shows the starting points on the rows and the arrival points on the columns (see Equation (7)). Each path is associated with a score that is the peak of the reference triangular membership functions.


(7) $$\begin{array}{ccccccccc} \mathrm{Start point}\mathrm{End Point} & \mathrm{Entrance} & \mathrm{Changing room} & \mathrm{Warehouse} & \mathrm{Break room} & \mathrm{Reporting room} & \mathrm{Recovery room} & \mathrm{Sterilization room} & \mathrm{Operating room}\\ \mathrm{Entrance} & 0 & 0 & 1 & 2 & 0 & 3 & 3 & 3\\ \mathrm{Changing room} & 0 & 0 & 3 & 0 & 0 & 0 & 0 & 0\\ \mathrm{Warehouse} & 0 & 0 & 0 & 0 & 0 & 0 & 0 & 1\\ \mathrm{Break room} & 0 & 0 & 0 & 0 & 0 & 0 & 0 & 1\\ \mathrm{Reporting room} & 0 & 0 & 0 & 0 & 0 & 0 & 0 & 0\\ \mathrm{Recovery room} & 0 & 0 & 0 & 0 & 0 & 0 & 0 & 1\\ \mathrm{Sterilization room} & 0 & 0 & 0 & 0 & 0 & 0 & 0 & 1\\ \mathrm{Operating room} & 0 & 0 & 0 & 0 & 0 & 0 & 0 & 0 \end{array}$$

**Figure 7:**
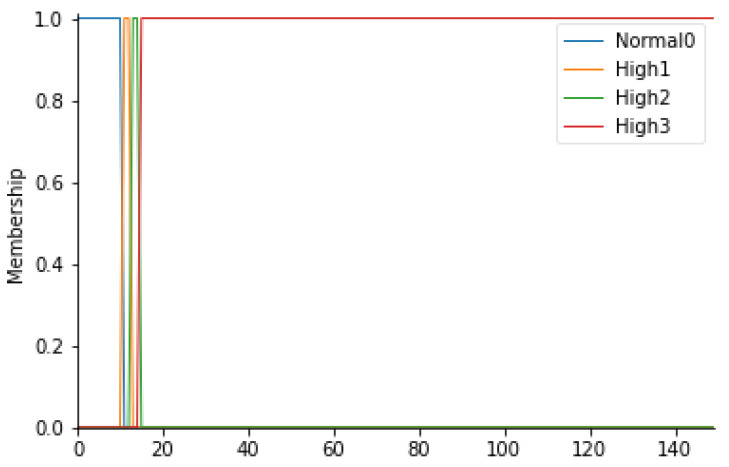
TPA membership functions.

By reporting the name of the areas in numerical form, it is possible for each user to access the matrix and assign the score to the path. The output produced is also a matrix showing the user id and the score associated with each point-to-point path for each row. Below is the section of code assigned to the calculation (Figure 7). A path where the temporal distance between the current area and the next is less than 10 min is considered continuous. Below (Algorithm 1) the code section written in pseudo-language used for the association of the score to the user path is reported.


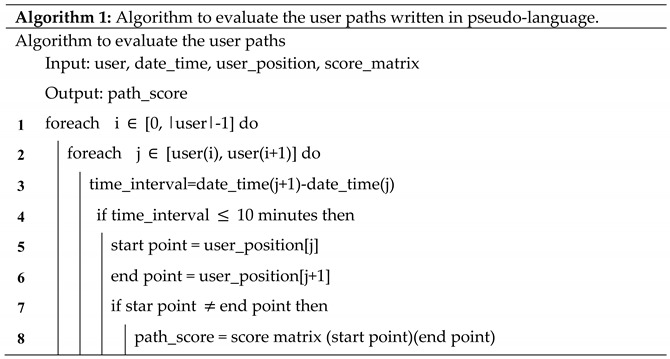

- Patient stay time in the prefilter area

In this study, the waiting time of the patient in the prefilter area (TPA) is an extremely important parameter. In fact, it is important that the patient transported from relative departments does not stay longer than the necessary time in the non-sterile area, an area in which a patient who has to undergo surgery could run into high risks of infection. Normal waiting time values are less than 10 min. If the wait settles for times exceeding the limit, the level of risk increases.

For this parameter, therefore, 4 fuzzy sets have been considered: Normal0, High1, High2, and High3. In detail, it is possible to consult Table 9 to observe the ranges considered. In Figure 7, the trapezoidal membership functions related to the various fuzzy sets can be observed.

- Definition of risk groups

**Figure 8:**
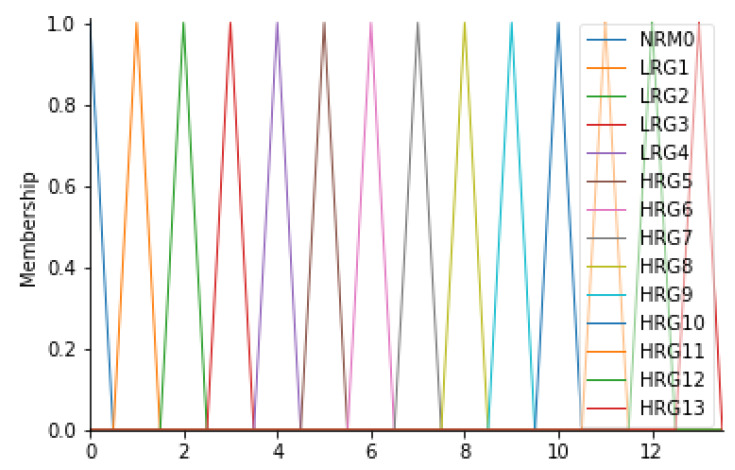
RG membership functions.

The output of the inference engine consists of the “Risk Group” variable (RG). This variable was found to be the most suitable for the case in question since it represents the level of risk associated with the risk of infection for the patient. In fact, this variable can assume 14 different values, on a scale from 0 to 13, where 0 is the level associated with a zero-risk level, while 13 is a very serious risk level; levels 1 to 12 represent an intermediate state of severity. Overall, therefore, there will be 14 fuzzy sets, signed with the L (low) in the case of a low-risk level, with the H (high) in the case of a high-risk level: NRM0, LRG1, LRG2, LRG3, LRG4, HRG5, HRG6, HRG7, HRG8, HRG9, HRG10, HRG11, HRG12, and HRG13. For this variable, it was considered appropriate to consider triangular membership functions. Table 10 and Figure 8 show the details of the membership functions with their respective ranges.

#### 2.3.5. Fuzzy Rules

**Figure 9:**
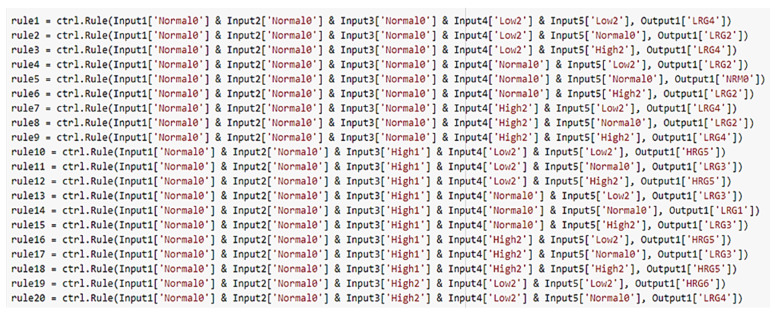
Fuzzy rules in Python.

The system developed in this work includes 576 rules covering all possible combinations between the input variables considered. Note that, the number of rules can be obtained from the following formula [44]:

$$\left[N=p_{1}\;x\;p_{2}\;x\ldots x\;p_{n}\right]$$

where *N* is the total number of possible rules, *n* is the number of linguistic variables, and *p_n_* is the number of linguistic terms for each linguistic variable. An extract of the rules (Figure 9) is shown below:

1. If Input1 = ‘Normal0’ & Input2 = ‘Normal0’ & Input3 = ‘Normal0’ & Input4 = ‘Low2’ & Input5 = ‘Low2’ then Output1 = ‘LRG4’
2. If Input1 = ‘Normal0’ & Input2 = ‘Normal0’ & Input3 = ‘Normal0’ & Input4 = ‘Low2’ & Input5 = ‘Normal0’ then Output1 = ‘LRG2’

.

.

.

572. If Input1 = ‘High3’ & Input2 = ‘High3’ & Input3 = ‘High3’ & Input4 = ‘High2’ & Input5 = ‘Normal0’ then Output1 = ‘HRG11’
573. If Input1 = ‘High3’ & Input2 = ‘High3’ & Input3 = ‘High3’ & Input4 = ‘High2’ & Input5 = ‘High2’ then Output1 = ‘HRG13’

#### 2.3.6. Defuzzification

Through the final defuzzification process, the combined fuzzy set from the aggregation process will output a single scalar quantity. Depending on the numerical value assumed by the system output, a warning message will show the need for urgent action based on the gravity of monitored parameters.

## 3. Results

The validation of the proposed strategy has been done offline by exploiting data collected via the devices installed in the OD of “Umberto I” Hospital situated in Nocera Inferiore, Salerno (Italy). In order to disclose the effectiveness of the proposed strategy, we have validated the FIS in two distinct scenarios. The aim of the evaluation of the FIS has been to verify experimentally whether according to a variation of the input, reflecting a contamination risk of the OR’s environmental conditions, the same output variation reflecting the input condition may be appreciated.

In what follow we will describe the results obtained from the validation of the fuzzy system for two different contamination levels detected for the OD:

- Case study 1: low risk of contamination;
- Case study 2: intermediate contamination risk, which represents a slightly compromised environmental picture.

### 3.1. Case Study 1

As the first case, parameters belonging to an OD environment status characterized by normal values except for the particle count have been considered. In particular, the situation is represented by the following parameters:

- Particle count: 3.583;
- OR Temperature: 20 °C;
- Relative humidity: 40%;
- Correctness of healthcare workers’ path: 1;
- Patient stay time in the prefilter area: 10 min.

These values represent a situation to be kept under control due to the value of the particle count, which is higher than the limit allowed by the standard. After implementing fuzzy rules, the analysis ends with the display of a message that correctly predicts the severity of the OD contamination status and the risk level obtained from defuzzification. Below the result of the code implemented with the indicated values is reported.

**Figure 10:**
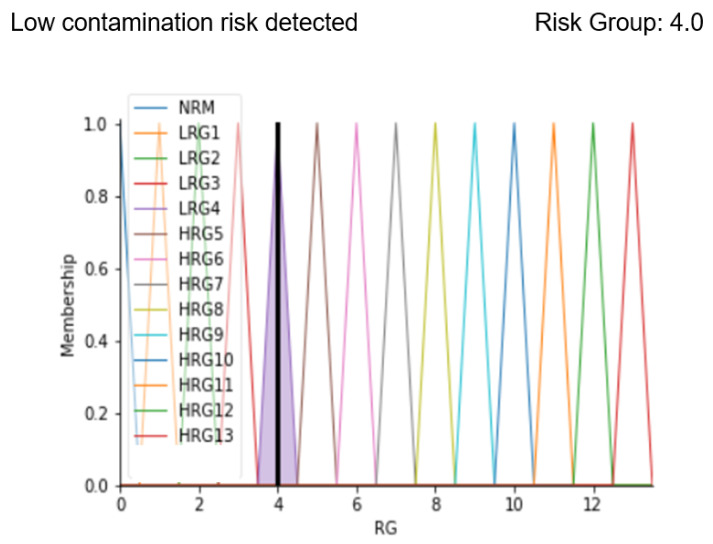
Results display for case study 1.

Results in Figure 10 show the membership function of the risk group highlighted with a black line corresponding to the calculated risk level. The analysis shows that there is low risk detected (in fact the message “Low contamination risk detected” is displayed) and the identified risk group is 4.0. After entering our code in a friendly interface, our system allows making the output of the system easier to be interpreted by both the patient and the clinician, since the output membership function graph and the result of the defuzzification process are clearly displayed.

### 3.2. Case Study 2

As the second example, the case of a compromised environment condition has been considered. In fact, both the particle count and correctness of healthcare workers’ path values are altered as follows:

- Particle count: 3.704;
- OR Temperature: 20 °C;
- Relative humidity: 50%;
- Correctness of healthcare workers’ path: 2;
- Patient stay time in the prefilter area: 13 min.

**Figure 11:**
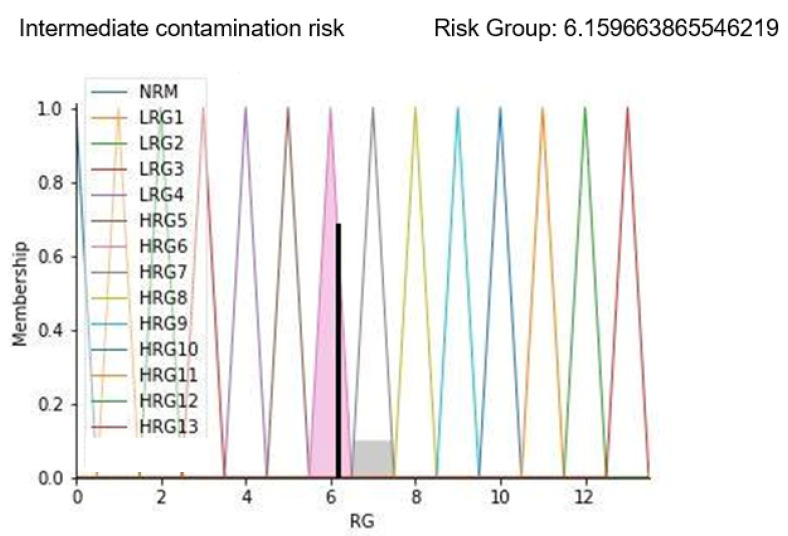
Results display for case study 2.

With these parameters, it is possible to highlight that a wrong healthcare workers’ path can negatively affect the environmental contamination status. In fact, this aspect can only have a negative impact on FIS implementation results by increasing the value of the risk group. Results are shown in Figure 11.

Results in Figure 11 show the membership function of the risk group highlighted with a black line corresponding to the calculated risk level. As can be seen from the displayed alarm, the risk group value is increased to more than 6; this is confirmed by the sentence “Intermediate contamination risk” displayed as a result. On a scale of 13 levels, this predicted value represents an environment with a concrete risk of contamination that could degenerate if timely corrective actions are not applied.

Despite the appreciable results, the proposed methodology has some shortfalls. The apparatus used to reveal the input parameters of the FIS (the number of particles in the air, temperature, humidity, number of personnel in a room) were not capable of determining which and how many of the aerosolized particles were live bacteria. However, as discussed in [4], particle concentration correlates with the concentration of viable bacteria during the corresponding period. Further, following on from this, we did not correlate OR air quality with an eventual increase in infection incidence. Performing a clinical study to demonstrate this obviously would not be ethically acceptable for patients. Another weakness is the need to reconfigure all rules in case a new parameter would be taken into account in order to monitor air quality. Nevertheless, results demonstrate that the designed FIS returns a reliable estimate of the actual situation of the OD’s environmental quality. The advantage of the proposed system is that it is configured as a DSS (decision support system) helping clinicians and managers to evaluate OD’s air quality on the basis of a set of rules using heterogeneous parameters.

## 4. Discussion

This paper proposes a fuzzy inference system for the identification and the determination of the risk level related to the air quality in the OD in order to prevent surgical site infections (SSI). The aim is to provide a means through which early detect contamination risk and prevent critical situations both for clinicians and patients.

To check the correct behavior of the system, real inputs were considered. Appropriate devices—i.e., BLE devices, a particle counter, and a multi-parameter instrument—were installed in the OD of the “Umberto I” Hospital in Nocera Inferiore (Italy), seat of the experimental part of the BIPS project. The detection activities carried out have made it possible to achieve the objectives set.

To include all possible inputs, 573 inference rules have been developed, mathematically formulated to allow the conversion of the fuzzy system output into a single value, attributed to the risk of contamination of the OD environment. Then some tests have been carried out, considering parameters for different situations. Two of them have been reported in order to show the high capability and precision of the FIS to discern situations that could be underestimated by a human assessment. The examples shown in the Results section are generalizable and applicable to the whole dataset.

Besides, another benefit for users of our FIS is that our system allows for easy presentation of the real conditions of air quality in OD with clear graphics and incisive alarm sentences. With the steady development of healthcare systems that take advantage of the potential of AI, this type of real-time system should play a key role in minimizing errors and enhancing the quality and efficiency of healthcare by making clinicians aware of the risk they are taking.

Future developments could implicate the consideration of more indicators than those considered in our paper in order to build an even better and more accurate system able to provide an increasingly global spectrum of the state of risk from contamination of operating rooms, which also includes the risk of COVID-19 contamination. The progressive deepening of the main indicators may lead to expanding the range of action and validating the methodology for studying outdoor air quality as well [45,46,47,48].

## 5. Conclusions

In this work, we propose a DSS based on a fuzzy inference system for monitoring the air quality in the OD in order to preserve the surgical environment and to optimize the consequent managing decisions, thus preventing SSI. The projected system works by processing a series of input data (i.e., OR temperature and relative humidity, the correctness of healthcare workers’ path, etc.) collected in real time through hi-tech sampling devices opportunely located. To validate the designed FIS, real inputs collected at the “Umberto I” Hospital situated in Nocera Inferiore, Salerno (Italy), were considered. In the case studies presented, our system proves to return a reliable estimate of the situation of the OD’s air quality; it returns risk values with extreme precision. The limitation in terms of type and number of inputs can represent an extremely interesting starting point for future analyses to support risk management in the ODs.

## Figures and Tables

**Table 1:** Data collected by BLE devices.

Type of Data	Format	Measuring Range	Unit of Measure
Data	Data	dd/mm/yyyy	-
Hour	Hour	hh:mm:ss	-
Echo Beacon ID	Number	aa:bb:cc:dd:ee:ff	-
Beacon ID	Number	aa:bb:cc:dd:ee:ff	-
RSSI value	Number	−120/0	dBm

**Table 2:** Data collected by the particle counter.

Type of Data	Format	Measuring Range	Unit of Measure
Data	Data	dd/mm/yyyy	-
Hour	Hour	hh:mm:ss	-
Particle Count (0.5 µm)	Number	35.300.000	particles/m^3^

**Table 3:** Data collected by the multi-parameter tool.

Type of Data	Format	Measuring Range	Unit of Measure
Data	Data	dd/mm/yyyy	-
Hour	Hour	hh:mm:ss	-
Temperature	Number	−40/+150	Celsius degree
Relative humidity	Number	0%/100%	Percentage

**Table 4:** Ranges of the particle count and their respective fuzzy sets.

Input	Range	Fuzzy Set
Particle count	<3.540	Normal0
	3.520–3.600	High1
	3.580–3.700	High2
	>3.680	High3

**Table 5:** Ranges of temperature and their respective fuzzy sets.

Input	Range	Fuzzy Set
Temperature (°C)	<20	Low2
	18–26	Normal0
	>24	High2

**Table 6:** Ranges of relative humidity and their respective fuzzy sets.

Input	Range	Fuzzy Set
Relative humidity	<40	Low2
	30–70	Normal0
	>60	High2

**Table 7:** Ranges of HWP and their respective fuzzy sets.

Input	Range	Fuzzy Set
Correctness of healthcare workers’ path	−0.5–0.5	Normal0
	0.5–1.5	High1
	1.5–2.5	High2
	2.5–3.5	High3

**Table 8:** Paths and their respective fuzzy sets.

Start Point	End Point	Fuzzy Set
Entrance	Operating room	High3
Entrance	Sterilization room	High3
Entrance	Break room	High2
Entrance	Recovery room	High3
Entrance	Warehouse	High1
Sterilization room	Operating room	High1
Changing room	Operating room	High3
Warehouse	Operating room	High1
Recovery room	Break room	High1
Break room	Operating room	High1

**Table 9:** Ranges of TPA and their respective fuzzy sets.

Input	Range	Fuzzy Set
TPA	<11	Normal0
	10–13	High1
	12.5–15.5	High2
	>15	High3

**Table 10:** Ranges of RG and their respective fuzzy sets.

Output	Range	Fuzzy Set
RG (risk group)	0 < RG < 0.5	NRM
	0.5 < RG < 1.5	LRG1
	1.5 < RG < 2.5	LRG2
	2.5 < RG < 3.5	LRG3
	3.5 < RG < 4.5	LRG4
	4.5 < RG < 5.5	HRG5
	5.5 < RG < 6.5	HRG6
	6.5 < RG < 7.5	HRG7
	7.5 < RG < 8.5	HRG8
	8.5 < RG < 9.5	HRG9
	9.5 < RG < 10.5	HRG10
	10.5 < RG < 11.5	HRG11
	11.5 < RG < 12.5	HRG12
	12.5 < RG < 13.5	HRG13

## Data Availability

The datasets generated and/or analyzed during the current study are not publicly available for privacy reasons but could be made available from the corresponding author on reasonable request.

## Abbreviations

BIPS	“Bacterial Infections Post Surgery” Project
OR	Operating room
OD	Operating department
HAI	Healthcare-associated infections
SSI	Surgical site infections
FL	Fuzzy logic
MF	Membership function
FES	Fuzzy expert system
FIS	Fuzzy inference system
FLC	Fuzzy logic controller
COG	Centre of gravity
HVAC	Heating, ventilation, and air conditioning
EIAQI	Environment indoor air quality index
HWP	Healthcare workers path
IAP	Indoor air pollutants
TCP	Thermal comfort pollutants
PC	Particle count
RG	Risk group
RH	Relative humidity
RSSI	Received signal strength indicator
T	Temperature
TPA	Time in the pre-filter area
DSS	Decision support system
